# Reliability and validation of the German WHOQOL-BREF in adults with congenital heart disease

**DOI:** 10.1007/s11136-026-04253-5

**Published:** 2026-05-03

**Authors:** Dominik Schröder, Lisa Nebel, Timothy R. Le Butt, Monika Sadlonova, Christoph Herrmann-Lingen, Ulrich Krause, Daniel Broschmann, Claudia Dellas

**Affiliations:** 1 Department of General Practice, University Medical Center Göttingen, Göttingen, Germany; 2 Department of Psychosomatic Medicine and Psychotherapy, University Medical Center Göttingen, Göttingen, Germany; 3 Department of Cardiac, Thoracic and Vascular Surgery, University Medical Center Göttingen, Göttingen, Germany; 4 Department of Geriatrics, University Medical Center Göttingen, Göttingen, Germany; 5 German Center for Cardiovascular Research (DZHK), Partner Site Lower Saxony, Göttingen, Germany; 6 Division of Pediatric Cardiology and Intensive Care Medicine, Department of Pediatrics and Adolescent Medicine, Center for Adults with Congenital Heart Disease, University Medical Center, Göttingen, Germany

**Keywords:** Quality of life, Congenital heart disease, Validation, WHOQOL-BREF, Quantitative methodology

## Abstract

**Purpose:**

Quality of Life (QoL) is a key outcome in adults with congenital heart disease (ACHD). While the WHOQOL-BREF is broadly applied, its psychometric properties have not been systematically assessed in German-speaking ACHD. This study aimed to evaluate the reliability and validity of the German WHOQOL-BREF in this population.

**Methods:**

Descriptive statistics and item characteristics were calculated. Internal consistency was examined using Cronbach’s α and item-rest correlations. Construct validity was assessed with confirmatory factor analysis (CFA) of the original four-domain model. Known-groups validity was evaluated by comparing QoL scores across NYHA class, arrhythmia, and heart failure status. Associations between WHOQOL-BREF domains and the global items of quality of life and general health were examined using regression models.

**Results:**

A total of 846 ACHD patients participated (mean age = 33.08 ± 12.0 years, 45.3% female); after excluding cases with excessive missing responses, 805 remained for analysis. Internal consistency ranged from acceptable to good (Cronbach’s α = 0.69–0.84). CFA confirmed the four-factor structure with good model fit (CFI = 0.993, RMSEA = 0.036, SRMR = 0.045). Known-groups comparisons showed lower QoL scores in patients with higher symptom burden. Regression analyses demonstrated that psychological and physical domains were the strongest factors of global QoL and health, explaining up to 47.4% and 44.8% of the variance.

**Conclusion:**

The German WHOQOL-BREF demonstrates good psychometric properties in ACHD and provides a robust tool for multidimensional QoL assessment, though the social domain shows limitations.

**Supplementary Information:**

The online version contains supplementary material available at 10.1007/s11136-026-04253-5.

## Background

Quality of life (QoL) is a well-established and widely used patient-reported outcome in clinical research. Contemporary frameworks acknowledge QoL as a multidimensional construct reflecting patients’ subjective well-being beyond traditional biomedical outcomes [1, 2]. In individuals with congenital heart disease (CHD) – a group of structural heart defects present at birth — QoL has gained particular relevance [3–5]. Due to lifelong medical surveillance, repeated interventions and psychosocial challenges, patient-reported outcomes provide essential information beyond biomedical markers.

Recent studies in this population highlight a bidirectional relationship between clinical status and QoL, underlining its prognostic relevance: clinical indicators such as defect complexity [6, 7], the number of cardiac diagnoses [7, 8], surgical history [9], and functional status [6, 7, 9, 10] have been identified as key predictors of QoL. Worse QoL has been linked to increased mortality, higher rates of hospitalization, and greater need for advanced therapies in adults with congenital heart disease (ACHD) and heart failure [11]. Beyond clinical variables, socio-demographic characteristics have likewise been identified as significant determinants of QoL, including education, employment and financial resources, and social support [6, 7, 12, 13]. Notably, while individuals with more complex CHD tend to report lower functional QoL [6–8]—referring to health-related QoL (HRQoL) measures that primarily capture the absence of pain and physical impairment—overall life satisfaction often remains stable or even high [6, 14]. This phenomenon is widely described in chronic illness research as the “well-being paradox” [15].

This highlights the conceptual distinction between HRQoL and broader subjective QoL, which has repeatedly been emphasized in QoL research (e.g., Karimi and Brazier [16]). Functional HRQoL measures operationalize high QoL in terms of the absence of pain and impairment, thereby reflecting an underlying hedonic conceptualization of QoL. The World Health Organization (WHO) defines QoL in a more eudaemonic manner as a person’s subjective evaluation of their life circumstances, shaped by cultural context, personal goals, expectations, and social values [17]. With the WHOQOL-BREF, the WHO seeks to translate this comprehensive concept into a brief, internationally applicable instrument. Unlike health-focused measures, it explicitly incorporates diverse aspects of psychological well-being, social relationships, and environmental conditions [18].

This broader conceptualization may be particularly relevant for ACHD. Living with CHD from birth, they face unique developmental and psychosocial challenges—such as the transition from pediatric to adult care, family planning, and aging with a chronic congenital condition [19–22]. These experiences may influence how individuals evaluate their QoL in ways that are not fully captured by health status alone.

Although the WHOQOL-BREF, by virtue of the conceptual framework underlying its operationalization, appears suitable for addressing the specific characteristics of the ACHD population, and it has been validated in numerous populations and languages, it [23] has not yet been systematically validated in German-speaking ACHD populations. However, whereas the SF-36, a HRQoL measure, is the most frequently used in this population the WHOQOL-BREF ranks fifth among the most commonly applied QoL instruments [24]. Given the specific medical, developmental, and psychosocial characteristics of this group, and considering that the WHOQOL-BREF’s psychometric properties are known to vary across clinical [25] and age groups [22], population-specific validation is warranted to ensure appropriate interpretation and use. Therefore, the present study aims to evaluate the psychometric properties of the German version of the WHOQOL-BREF in a large sample of ACHD, and to assess its suitability for assessing subjective QoL in this distinct population.

## Methods

### Study design and sample

The GoEMAH registry (*Göttinger EMAH* Register) was established in 2015 at the ACHD center of the Department of Pediatric Cardiology and Intensive Care Medicine at the University Medical Center Göttingen and has consecutively included all ACHD patients from the outpatient clinic up to 2019, who gave their written consent for participation. The study was approved by the local ethics committee of the University Medical Center Göttingen (approval number: 11/3/15). The registry contains detailed information on patients’ age, sex, level of education, diagnoses, medical history, previous surgeries and interventions, current medication, laboratory results, ECG findings, cardiopulmonary exercise testing, echocardiography, MRI, and cardiac catheterization results obtained at the time of inclusion or extracted from past medical records. In addition, patients were asked to complete the German version of the WHOQOL-BREF on the date of study inclusion. The classification of CHD to mild, moderate and severe complexity followed the ESC guidelines 2020 [26]. For patients with multiple CHD diagnoses, the defect with the highest complexity was defined as the leading diagnosis.

### Measure—WHOQOL-BREF

The WHOQOL-BREF is a concise self-report questionnaire developed by an international WHOQOL Group coordinated by the WHO to assess QoL, comprising 26 items in total: the first two items measure overall QoL and health (analyzed separately), while the remaining 24 items are distributed across four domains (physical, psychological, social, and environmental) on a five-point Likert-type response scale [27, 28]. Three items must be reverse scored so that higher values uniformly represent better perceived QoL. If more than 20% of the 26 items (six or more items) are missing, the participant is excluded. Otherwise, domain-level imputation is permitted so that in the physical, psychological and environmental domains, up to two missing items can be replaced by the mean of the remaining items (if three or more items are missing, no score is calculated for this domain). In the social domain, one missing item is permitted. Raw domain scores are obtained by calculating the mean of the items of the domain (after handling any missing responses and reverse-scoring), then multiplied by four (in order to make domain scores comparable with the scores used in the WHOQOL-100) and transformed linearly to a 0–100 possible range, with higher scores reflecting better perceived QoL. The WHOQOL-BREF has undergone extensive worldwide validation, demonstrating reliable cross-cultural applicability, and full administration and scoring instructions are available in the WHOQOL-BREF manual published by the WHO [27].

### Data analysis

All analyses were conducted using R (version 4.4.0) using the packages lavaan [29], psych [30] and ggplot2 [31]. Prior to analysis, item responses were checked for plausibility by verifying that all values fell within the valid response range of the WHOQOL-BREF (1–5) and recoded according to WHOQOL-BREF guidelines [18]. Items Q3, Q4, and Q26 were reverse coded. Cases with six or more missing items were excluded, in line with the manual's recommendation. For the remaining cases, missing values were imputed within domains if the number of missing items did not exceed predefined thresholds (≤ 2 for physical, psychological, and environment domains; ≤ 1 for the social domain). Imputation was performed using the individual mean of available items within each domain.

#### Item analysis and reliability

Descriptive statistics were computed for raw scores of all items, including means, standard deviations, proportion of missing values, floor and ceiling effects. Internal consistency reliability of the domains was assessed using Cronbach’s alpha. Item-rest correlations and Cronbach’s alpha if an item was deleted was calculated to evaluate the contribution of each item to domain reliability.

#### Scale distribution

Mean, SD and skewness and excess kurtosis of the domains and global items were reported. To assess scale distribution characteristics, Shapiro–Wilk tests for normality were performed.

#### Correlations between domains and global items

To assess bivariate association between the domains and global items, spearman and Pearson correlation coefficients between all domains and both global items were calculated.

#### Construct validity and known group validity

Construct validity was tested using confirmatory factor analysis (CFA) for the four-factor structure of the WHOQOL-BREF, applying the WLSMV estimator. CFA was used to test the established four-domain structure of the WHOQOL-BREF, as the present study aimed to evaluate whether this existing model applies to ACHD. Model fit was evaluated using multiple indices with cut-offs for a good model fit based on Ximenez et al. [32] including the chi-square statistic, comparative fit index (CFI) (> 0.95), Tucker-Lewis index (TLI) (> 0.95), root mean square error of approximation (RMSEA) (< 0.05) with its 90% confidence interval (CI), and standardized root mean square residual (SRMR) (< 0.08). As sensitivity analyses, a unidimensional model and a bifactor model (including a general QoL factor and four domain-specific factors) were additionally estimated to examine whether an alternative factor structure would provide a better model fit. Second, known-groups validity was examined by comparing WHOQOL-BREF scores across clinically relevant subgroups defined by New York Heart Association (NYHA) class, heart failure diagnosis, and cardiac arrhythmia diagnosis. These variables were selected because previous research has consistently identified NYHA class as a significant determinant of HRQoL in ACHD [6, 7, 10], while heart failure and arrhythmias are well-established contributors to reduced HRQoL in patients with acquired heart disease [33, 34]. Although evidence in the ACHD population is limited, available studies suggest that heart failure [7, 35] and arrhythmias [36] are also associated with impaired HRQoL in this group. For each subgroup, mean scores and 95% CIs were calculated and visualized for all WHOQOL-BREF domains and global items. Group differences across NYHA classes were tested using one-way ANOVA with effect sizes reported as eta-squared and followed by Bonferroni-corrected pairwise t-tests. Comparisons for heart failure and cardiac arrhythmias were conducted using independent-samples t-tests. Demonstrating expected differences between these clinical groups provides further support for the construct validity of the WHOQOL-BREF in this population.

#### Influence of WHOQOL-BREF domains on general QoL and general health

To examine the association between WHOQOL-BREF domains and the two global items (overall quality of life and general health), linear regression models were computed with the global items as dependent variables. As independent variables all four domains were included into the models to identify the strongest factors of global QoL and general health.

## Results

A total of 846 patients were recruited between 2015 and 2019. Of these, 31 were excluded due to having six or more missing items on the WHOQOL-BREF. An additional 10 patients were excluded for exceeding the allowable number of missing values within at least one domain, as specified in the WHOQOL-BREF manual. Consequently, data from 805 patients were included in the final analysis.

### Sample characteristics

The study included 805 adults with CHD (54.7% male; mean age 33.1 ± 12.0 years). More than half of the participants were between 18 and 30 years old (51.8%). According to the European Society of Cardiology (ESC) severity score, the majority had moderate disease complexity (48.2%). The most frequent main diagnosis was right ventricular outflow tract obstruction (21.0%), and most patients were classified as NYHA I (63.4%). Further sample characteristics are presented in Table 1.

**Table 1:** Sample characteristics

N = 805	n/mean (%/SD)
*Sex*	
Male	440 (54.7)
Female	365 (45.3)
Age	33.08 (12.0)
18–30	417 (51.8)
31–50	306 (38.0)
51–65	76 (9.4)
65 +	6 (0.7)
*ESC Severity Score*	
Mild	215 (26.7)
Moderate	388 (48.2)
Severe	128 (15.9)
Not classified	74 (9.2)
*NYHA classification*	
I	510 (63.4)
II	239 (29.7)
III	44 (5.5)
IV	6 (0.7)
Missing	6 (0.7)
*Arrhythmia*	
Yes	320 (39.8)
No	469 (58.3)
Missing	16 (2.0)
*Heart failure*	
Yes	37 (4.6)
No	736 (91.4)
Missing	32 (4.0)
*Depression*	
Yes	37 (4.6)
No	766 (95.2)
Missing	2 (0.2)
BMI, kg/m^2^	25.84 (5.26)
Underweight (< 18.5)	27 (3.4)
Normal weight (18.5—< 25)	361 (44.8)
Pre-obesity (25—< 30)	248 (30.8)
Obesity Class I (30- < 35)	107 (13.3)
Obesity Class II & III (> 35)	62 (7.7)
*School education*	
No school education	52 (6.5)
Lower secondary education	121 (15.0)
Intermediate secondary education	448 (55.7)
Tertiary education	131 (16.3)
Missing	13 (1.6)
*Smoking status*	
Smoker	108 (13.4)
Ex-smoker	38 (4.7)
Non-smoker	618 (76.8)
Missing	41 (5.1)

Depression was self-reported; ESC: Severity Score according to Baumgartner et al. [26]; Ex-smoker was defined as not smoking for at least 6 months

BMI, Body-Mass-Index; ESC, European Society of Cardiology; NYHA, New York Heart Association functional class; SD, Standard deviation

### Item analysis and reliability

Table 2 summarizes item-level descriptive statistics, floor and ceiling effects, missing data before imputation, item-rest correlations for all WHOQOL-BREF domains and individual items. Item-rest-correlation and Cronbach’s α if the individual item would be deleted is reported for all items excluding the global items.

**Table 2:** Item-level descriptive statistics, item-rest correlations, and internal consistency reliability of WHOQOL-BREF domains before imputation

	Cronbach’s α (if item deleted on individual items)	Floor	Ceiling	Mean score raw (SD)	Item-rest correlation	Missings before imputation (%)
*Global Items*						
QoL (Q1)^a^	–	0.25	21.75	3.99 (0.72)	–	0.74
Health (Q2)^a^	–	1.76	13.07	3.64 (0.91)	–	1.22
Physical	0.84					
Pain and discomfort (Q3)^b^	0.81	0.62	59.88	4.40 (0.88)	0.59	0.49
Medical treatment (Q4)^b^	0.83	2.98	56.02	4.24 (1.07)	0.48	1.23
Energy (Q10)^c^	0.80	0.62	29.44	4.01 (0.84)	0.67	0.12
Discomfort (Q15)^a^	0.81	0.25	54.53	4.37 (0.81)	0.65	0.49
Sleep (Q16)^d^	0.85	2.24	16.89	3.59 (1.01)	0.37	0.00
Ability to perform daily living activities (Q17)^d^	0.79	0.75	31.06	4.05 (0.84)	0.73	0.37
Capacity for work (Q18)^d^	0.79	3.60	27.95	3.90 (1.01)	0.71	2.82
Psychological	0.79					
Positive feelings (Q5)^b^	0.74	0.37	30.81	4.08 (0.79)	0.63	0.74
Eudemonic well-being (Q6)^b^	0.76	0.75	48.70	4.32 (0.81)	0.55	1.47
Thinking, learning, memory and concentration (Q7)^b^	0.78	0.62	16.65	3.78 (0.81)	0.46	0.00
Bodily image and appearance (Q11)^c^	0.77	0.62	35.65	4.12 (0.82)	0.48	0.00
Satisfaction with self (Q19)^d^	0.73	1.12	17.89	4.87 (0.79)	0.67	1.35
Negative feelings (Q26)^e^	0.77	1.24	16.65	3.62 (0.94)	0.49	0.74
Social	0.69					
Personal relationships (Q20)^d^	0.50	0.99	35.90	4.09 (0.89)	0.58	0.74
Sexual activity (Q21)^d^	0.59	4.35	23.73	3.70 (1.06)	0.52	6.87
Social support (Q22)^d^	0.68	0.62	36.77	4.15 (0.82)	0.44	1.60
Environment	0.75					
Freedom, physical safety and security (Q8)^b^	0.72	0.12	24.47	4.01 (0.74)	0.49	1.23
Physical environment (Q9)^b^	0.74	0.00	29.94	4.11 (0.73)	0.38	1.23
Financial resources (Q12)^c^	0.73	2.24	28.82	3.86 (1.00)	0.44	0.12
Opportunities for acquiring new information and skills (Q13)^c^	0.71	0.00	57.89	4.50 (0.66)	0.55	0.25
Participation in and opportunities for recreation/leisure (Q14)^c^	0.72	0.62	37.89	4.00 (0.97)	0.47	0.61
Home environment (Q23)^d^	0.74	0.99	39.63	4.17 (0.88)	0.36	1.35
Health and social care: accessibility and quality (Q24)^d^	0.72	0.75	28.45	4.04 (0.79)	0.46	2.84
Transport (Q25)^d^	0.72	0.75	43.23	4.24 (0.83)	0.48	2.21

^a^rated on a 5-point-Likert-type response scale from “very poor” to “very good”; ^b^rated on a 5-point-Likert-type response scale from “not at all” to “extremely”; ^c^rated on a 5-point-Likert-type response scale from “not at all” to “completely”; ^d^rated on a 5-point-Likert-type response scale from “very dissatisfied” to “very satisfied”; ^e^rated on a 5-point-Likert-type response scale from “never” to “always”

Mean item scores ranged from 3.59 to 4.50 (SDs 0.66–1.07). Median, Q25 and Q75 of items scores are reported in Supplementary Table 1. Item-rest correlations varied between 0.36 and 0.73. Cronbach’s α ranged from 0.69 to 0.84 across domains. Only the exclusion of Item Q16 would lead to an improved Cronbach’s α. Floor effects were generally low (< 5%), while ceiling effects were more pronounced in several items (up to 59.9%). The proportion of missing values prior to imputation ranged from 0.0% to 6.9% (Q21 – Sexual activity).

### Scale distribution

Descriptive analyses of the WHOQOL-BREF domains and global items revealed average scores ranging from 66.05 (Global Health) to 77.88 (Environment). The standard deviations ranged from 12.58 to 22.73. All four domains and both global items showed negative skewness (– 1.00 to – 0.47), indicating that most patients reported relatively high levels of QoL. Excess kurtosis values ranged from – 0.16 to 1.00, suggesting no substantial deviations from normal kurtosis (Table 3). The Shapiro–Wilk tests for all scales were statistically significant (*p* < 0.001), suggesting deviations from a normal distribution.

**Table 3:** Descriptive statistics and distribution characteristics of WHOQOL-BREF domain scores and global items

	Mean	SD	Skewness	Excess kurtosis
Physical	77.01	16.47	− 1.00	0.80
Psychological	74.09	14.46	− 0.80	1.00
Social	74.50	18.21	− 0.77	0.66
Environment	77.88	12.58	− 0.47	− 0.16
Global QoL	74.66	18.01	− 0.58	0.78
Global Health	66.05	22.73	− 0.71	0.19

SD, Standard deviation; Shapiro–Wilk test *p* < 0.001 for all domains and global items

### Correlations between domains and global items

Spearman correlation coefficients between the WHOQOL-BREF domains and global items are presented in Table 4. Intercorrelations among domains ranged from *r* = 0.43 to 0.67. Correlations between domains and the global items ranged from *r* = 0.36 (Social–Health) to *r* = 0.62 (Psychological–QoL). Pearson correlations were similar to Spearman correlations.

**Table 4:** Pearson (above diagonal) and Spearman (below diagonal) correlations between WHOQOL-BREF domains and global items

Scale	Physical	Psychological	Social	Environment	QoL	Health
Physical	1	0.700	0.441	0.616	0.615	0.621
Psychological	0.669	1	0.522	0.632	0.641	0.606
Social	0.433	0.526	1	0.447	0.416	0.359
Environment	0.604	0.610	0.443	1	0.517	0.466
QoL	0.589	0.618	0.420	0.515	1	0.572
Health	0.560	0.558	0.358	0.453	0.548	1

QoL, Quality of life

### Construct validity

The four-factor model of the WHOQOL-BREF was evaluated using confirmatory factor analysis. Model fit was acceptable across all indices (Table 5). Estimated latent correlations between WHOQOL-BREF domains for the four-factor-model are reported in Supplementary Table 2. As sensitivity analyses, an unidimensional and a bifactor model were estimated, both of which showed poorer model fit across all indices compared with the four-factor model (Supplementary Table 3).

**Table 5:** Fit indices of the confirmatory factor analysis for the four-factor model of the WHOQOL-BREF

Model fit indices	Four-factor model
X^2^	620
df	246
X^2^/df	2.52
P (X^2^)	< 0.001
CFI	0.993
TLI	0.992
RMSEA (90% CI)	0.036 (0.032–0.039)
SRMR	0.045

CFI, Comparative Fit Index; CI, Confidence interval; df, Degrees of freedom; RMSEA, Root mean square error of approximation; SD, Standard deviation; SRMR, Standardized root mean square residual; TLI, Tucker–Lewis Index

**Fig. 1:**
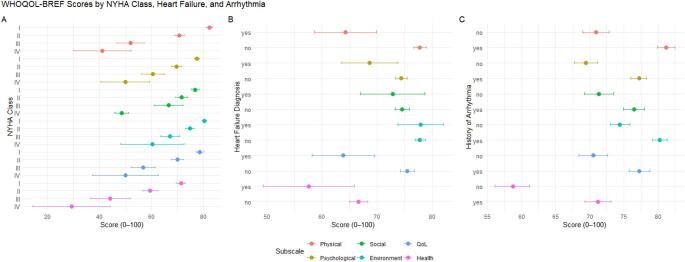
Mean and 95% CI of WHOQOL-BREF scores stratified after **A** NYHA class, **B** heart failure diagnosis and **C** cardiac arrhythmias diagnosis

Across all comparisons, patients with higher disease burden consistently reported worse health on most WHOQOL-BREF domains (Fig. 1). These differences were tested statistically by formal group comparisons, with significant overall effects across NYHA classes and clinically relevant differences for heart failure and cardiac arrhythmias (Supplementary Tables 4–6)

### Influence of WHOQOL-BREF domains on general QoL and general health

Multiple regression models including all four domains simultaneously showed that psychological and physical health were the strongest independent factors of both global outcomes. The models explained 47.4% of the variance in overall QoL and 44.8% in general health (Table 6).

**Table 6:** Multivariable linear regression models on global quality of life and global health from WHOQOL-BREF domains

Independent variable	Global QoL		Global Health	
	β (95% CI)	*p*	β (95% CI)	*p*
Physical	0.312 (0.229, 0.394)	< 0.001	0.521 (0.414, 0.628)	< 0.001
Psychological	0.438 (0.339, 0.536)	< 0.001	0.504 (0.377, 0.631)	< 0.001
Social	0.070 (0.010, 0.130)	0.021	0.027 (− 0.051, 0.104)	0.497
Environment	0.128 (0.028, 0.228)	0.012	0.042 (− 0.088, 0.173)	0.522
R^2^	0.474		0.448	

## Discussion

The present study examined the psychometric properties of the German version of the WHOQOL-BREF in a large sample of ACHD. Overall, participants reported relatively high levels of QoL. Interestingly, scores in the environmental domain suggest that many participants perceived contextual aspects of their lives—such as safety, access to health care, financial resources, and opportunities for leisure and mobility—as generally satisfactory. In contrast, lower scores were reported for global health and the psychological domain, indicating reduced positive affect, self-esteem, cognitive functioning, and body image. Across nearly all analyses, higher disease burden was consistently associated with lower scores across WHOQOL-BREF domains and global items, supporting the instrument’s known-groups validity (Fig. 1).

From a psychometric perspective, the WHOQOL-BREF demonstrated acceptable reliability and validity in this ACHD sample. Cronbach’s alpha coefficients for the four domains ranged from 0.69 (social) to 0.84 (physical), indicating good internal consistency, except for the social domain, which is consistent with findings in other populations [23, 37, 38]. Accordingly, results for the social domain should be interpreted with caution, particularly when applied at the individual level, given the limited number of items and the slightly lower internal consistency observed. CFA supported the four-factor structure with good model fit indices. All domains were significantly associated with the global items of QoL and general health, with the psychological and physical domains emerging as the strongest factors in both bivariate and multivariate regression analyses. These findings support the use of the WHOQOL-BREF as a valid and reliable tool to assess QoL in ACHD, while highlighting the limitations of the social domain due to its limited item count.

QoL research in CHD has expanded substantially since the 1990s, paralleling broader developments in patient-reported outcome measurement [24]. A wide range of instruments grounded in different conceptual frameworks is now available, including health-focused systems such as Patient-Reported Outcomes Measurement Information System (PROMIS®; e. g. Kaplan & Hays 2022 [39]) and broader QoL measures such as the WHOQOL. Rather than competing approaches, these instruments address complementary aspects of patient experience. However, the theoretical assumptions underlying these instruments are often insufficiently considered in their application, which may lead to misinterpretation of results [24].

Factor-analytic studies in diverse populations—such as Iranian patients with type 2 diabetes [40], older Brazilian women [41], and a representative Taiwanese sample [42]—have demonstrated that instruments like the SF-36 and WHOQOL-BREF assess overlapping but distinct underlying constructs. Notably, the SF-36 has been shown to be more sensitive to group differences related to health status, whereas the WHOQOL-BREF appears more responsive to variations in overall subjective QoL [42]. In the context of CHD—a lifelong condition that often affects long-term planning and the achievement of personal goals across multiple life domains such as employment, family life, and self-fulfillment—this distinction is clinically and conceptually relevant.

In the present study, the four WHOQOL-BREF domains explained 47.4% of the variance in overall QoL and 44.8% in general health. This finding is consistent with the theoretical premise that global QoL encompasses factors beyond the domains explicitly assessed by the instrument. For instance, qualitative research in young ACHD highlights values such as independence, normality, autonomy, and light-heartedness as central components of a good life that may not be fully represented in standard QoL domains [3, 4]. Our results align with findings by Palimaru and Hays, who reported that HRQoL measures accounted for 69% to 75% of the variance in overall QoL, highlighting the partial overlap between HRQoL and evaluative well-being [43]. However, their study was based on PROMIS instruments and US PROMIS samples drawn from the general population rather than from German ACHD. Moreover, the PROMIS instruments are grounded in conceptualizations of (HR)QoL that differ from those underlying the WHOQOL-BREF, and these conceptual as well as cultural and contextual differences may partly explain the differing proportions of explained variance. This indicates that the validity of QoL measures cannot be assumed to generalize to ACHD populations.

An interesting conceptual issue is the strong correlation between the psychological and physical domains with the overall QoL item. Environmental factors showed a weaker but still significant contribution, whereas the social domain demonstrated the smallest association. This finding may partly reflect measurement limitations, as the social domain is represented by only three items focusing on personal relationships, social support, and sexual activity. Additional relational dimensions—such as role fulfillment, partnership stability, or perceived normality in social participation—may be particularly relevant for ACHD and warrant further consideration in future research.

A key implication of our study is the appropriateness of the WHOQOL-BREF for use in ACHD while simultaneously illustrating domain-specific limitations, particularly within the social domain. Although the instrument has been extensively validated, accumulating evidence indicates that reliability, factor structure, and domain relevance may vary across clinical groups [38, 44–46].

The original WHOQOL-100 offers greater granularity, particularly in the social and environmental domains, but its length renders it less practical for routine clinical or research use. The WHOQOL-BREF provides a pragmatic alternative, but the brevity may compromise internal consistency and sensitivity to change [47]. Especially the social domain with its limited number of three items only restricts its ability to capture the full range of social functioning and may explain both the lower reliability coefficients and the higher proportion of missing responses, particularly for the item on sexual activity—a pattern consistently reported in other studies [48–51]. Moreover, handling of missing values varies widely across studies. The WHO manual recommends mean imputation within each domain if only one (social) or up to two items (other domains) are missing, yet other approaches (e.g., full information maximum likelihood [23]) are also used. A study has shown that including more items within the same domain for imputation improves accuracy, while using items from other domains is not particularly helpful; extreme response categories tend to be least accurately imputed [52]. In our study, missing values were imputed following the WHOQOL-BREF manual [27] using the individual mean of available items within the domain.

### Limitations

The study comes along with several limitations that should be considered when interpreting the results. The generalizability of the findings remains uncertain, as the data come from a single center in Germany. The study population is comparable to registries from other ACHD centers with regard to age, sex and severity of ACHD [53]. However, the distribution of CHD severity differs between ACHD centers caring for more patients with moderate/complex disease (approx. 60–70%) and the general ACHD population in Germany (40%) [8, 54]. The majority of participants in our cohort were classified as NYHA class I or II, while only few patients were assigned to higher NYHA classes. In particular, the very small number of patients in NYHA class IV limits the precision of estimates for this group and likely explains the wide confidence intervals observed. Therefore, findings from known-groups analyses should be interpreted with caution and cannot be readily generalized to ACHD patients with severe functional limitations.

In terms of psychometric evaluation, the study relied on confirmatory factor analysis, which demonstrated an excellent fit for the proposed four-factor structure of the WHOQOL-BREF. However, exploratory factor analysis was not performed. The reliability of the social domain was lower than that of the other domains, which is most likely attributable to the limited number of items in this domain. This pattern, including a higher proportion of missing answers for the item on sexual activity, is consistent with findings from previous research and reflects a known limitation of the instrument.

Furthermore, only the WHOQOL-BREF was used to assess QoL. The absence of additional instruments limits the examination of convergent validity, although known-groups validity was supported. As the study employed a cross-sectional design with only a single measurement per patient, neither test–retest reliability nor sensitivity to change over time could be evaluated. However, in a clinical sample, moderate to strong correlations have been observed between related health domains, with coefficients reaching up to approximately r = 0.67 [40]. In contrast, population-based analyses by Huang et al. (2006) demonstrated substantially weaker cross-instrument associations and clearly distinct factor structures [42]. Similarly, Castro et al. (2014) reported only weak convergent validity in older adult samples [41].

Finally, measurement invariance across different subgroups within the ACHD population was not investigated. This was beyond the primary scope of the present study, which aimed to evaluate the overall psychometric properties of the WHOQOL-BREF in an ACHD population rather than to examine subgroup-specific measurement structures. In addition, given the single-center design and the lack of guaranteed representativeness across clinical and demographic strata, subgroup analyses would require cautious interpretation. Therefore, potential differences between subgroups (e.g., by age, sex, or disease complexity) remain to be explored in future studies.

## Conclusions

The German WHOQOL-BREF is a valid and reliable tool for assessing QoL in ACHD. Its multidimensional structure was confirmed, with psychological and physical domains showing the strongest associations with overall QoL and health. While the measure is appropriate for clinical and research contexts, its application and interpretation should be guided by a careful reflection of the underlying conceptual model. Distinguishing between instruments grounded in a health-related functional perspective and those based on a broader concept of subjective well-being remains essential for meaningful comparison and interpretation. Therefore, future studies should not only address psychometric issues but also explicitly consider the theoretical assumptions that shape what QoL is understood to represent.

## Supplementary Information

Below is the link to the electronic supplementary material.

Supplementary Material 1

## Data Availability

The data that support the findings of this study are available from the corresponding author, CD, upon reasonable request.
