# Supplementary material for: Reliability and validation of the German WHOQOL-BREF in adults with congenital heart disease
Source: Qual Life Res. 2026 May 3;35(6):149. doi: 10.1007/s11136-026-04253-5 (PMC13136210; doi:10.1007/s11136-026-04253-5)
Supplement: Supplementary file 1 — Supplementary Material 1 [file 11136_2026_4253_MOESM1_ESM.docx]

# Supplement

Supplementary Table 1. Item-level distribution of WHOQOL-BREF responses (median and interquartile range)

|  | Median | Q25 | Q75 |
| --- | --- | --- | --- |
| Global Items |  |  |  |
| QoL (Q1)^1^ | 4 | 4 | 4 |
| Health (Q2)^1^ | 4 | 3 | 4 |
| Physical |  |  |  |
| Pain and discomfort (Q3)^2^ | 5 | 4 | 5 |
| medical treatment (Q4)^2^ | 5 | 4 | 5 |
| Energy (Q10)^3^ | 4 | 4 | 5 |
| discomfort (Q15)^1^ | 5 | 4 | 5 |
| Sleep (Q16)^4^ | 4 | 3 | 4 |
| ability to perform daily living activities (Q17)^4^ | 4 | 4 | 5 |
| capacity for work (Q18)^4^ | 4 | 4 | 5 |
| Psychological |  |  |  |
| Positive feelings (Q5)^2^ | 5 | 4 | 5 |
| Well-being (Q6)^2^ | 4 | 4 | 5 |
| Thinking, learning, memory and concentration (Q7)^2^ | 5 | 3 | 4 |
| Bodily image and appearance (Q11)^3^ | 4 | 4 | 5 |
| Satisfaction with self (Q19)^4^ | 4 | 3.8 | 4 |
| Negative feelings (Q26)^5^ | 4 | 4 | 5 |
| Social |  |  |  |
| Personal relationships (Q20)^4^ | 4 | 4 | 5 |
| Sexual activity (Q21)^4^ | 4 | 3 | 4.5 |
| Social support (Q22)^4^ | 3 | 4 | 4 |
| Environment |  |  |  |
| Freedom, physical safety and security (Q8)^2^ | 4 | 4 | 4.29 |
| Physical environment (Q9)^2^ | 4 | 4 | 5 |
| Financial resources (Q12)^3^ | 4 | 3 | 5 |
| Opportunities for acquiring new information and skills (Q13)^3^ | 5 | 4 | 5 |
| Participation in and opportunities for recreation/leisure (Q14)^3^ | 4 | 3 | 5 |
| Home environment (Q23)^4^ | 4 | 4 | 5 |
| Health and social care: accessibility and quality (Q24)^4^ | 4 | 4 | 5 |
| Transport (Q25)^4^ | 4 | 4 | 5 |

^1^rated on a 5-point- Likert-type response scale from “very poor” to “very good”; ^2^rated on a 5-point- Likert-type response scale from “not at all” to “extremely”; ^3^rated on a 5-point- Likert-type response scale from “not at all” to “completely”; ^4^rated on a 5-point- Likert-type response scale from “very dissatisfied” to “very satisfied”; ^5^rated on a 5-point- Likert-type response scale from “never” to “always”

Supplementary Table 2. Estimated latent correlations among WHOQOL-BREF domains

|  | Physical | Psychological | Social | Environment |
| --- | --- | --- | --- | --- |
| Physical | 1.00 |  |  |  |
| Psychological | 0.87 | 1.00 |  |  |
| Social | 0.58 | 0.71 | 1.00 |  |
| Environment | 0.79 | 0.84 | 0.62 | 1.00 |

Supplementary Table 3. Fit indices of the confirmatory factor analysis for the one-factor nad bifactor model of the WHOQOL-BREF

| Model fit indices | One-factor-model | Bifactor-model |
| --- | --- | --- |
| X^2^ | 1657 | 750 |
| df | 252 | 228 |
| X^2^/df | 6.58 | 3.29 |
| P (X^2^) | <0.001 | <0.001 |
| CFI | 0.786 | 0.904 |
| TLI | 0.765 | 0.884 |
| RMSEA (90% CI) | 0.100 (0.095 – 0.105) | 0.070 (0.065 – 0.076) |
| SRMR | 0.068 | 0.045 |

CFI: Comparative Fit Index; CI: Confidence interval; df: Degrees of freedom; RMSEA: Root mean square error of approximation; SD: Standard deviation; SRMR: Standardized root mean square residual; TLI: Tucker–Lewis Index

Supplementary Table 4. Mean and 95% CI of WHOQOL-BREF scores stratified after NYHA class

| NYHA Class / Domain | I | II | III | IV | Eta-squared | p^1^ |
| --- | --- | --- | --- | --- | --- | --- |
| Physical | 82.4 (81.3; 83.5) | 70.8 (68.9; 72.8) | 52.0 (46.7; 57.2) | 41.1 (30.0; 52.2) | 0.274 | <0.001 |
| Psychological | 77.6 (76.5; 78.7) | 69.7 (67.8; 71.6) | 60.5 (56.1; 65.0) | 50.0 (40.6; 59.4) | 0.135 | <0.001 |
| Social | 76.9 (75.4; 78.4) | 71.6 (69.3; 73.9) | 66.7 (61.1; 72.2) | 48.6 (45.9; 51.3) | 0.044 | <0.001 |
| Environment | 80.4 (79.5; 81.4) | 74.8 (73.1; 76.5) | 67.2 (63.7; 70.7) | 60.4 (48.3; 72.5) | 0.099 | <0.001 |
| QoL | 78.7 (77.2; 80.2) | 70.0 (67.8; 72.3) | 56.8 (52.2; 61.4) | 50.0 (37.3; 62.7) | 0.120 | <0.001 |
| Health | 71.4 (69.6; 73.1) | 59.5 (56.7; 62.4) | 44.2 (36.6; 51.8) | 29.2 (14.1; 44.2) | 0.130 | <0.001 |

^1^P-values derived from one-way ANOVA

Supplementary Table 5. Pairwise comparisons of WHOQOL-BREF domain scores between NYHA classes (independent group t-tests, Bonferroni-corrected p-values)

|  | NYHA I vs. II | NYHA II vs. III | NYHA I vs. IV | NYHA II vs. NYHA III | NYHA II vs. NYHA IV | NYHA III vs NYHA IV |
| --- | --- | --- | --- | --- | --- | --- |
| Physical | <0.001 | <0.001 | <0.001 | <0.001 | <0.001 | 0.450 |
| Psychological | <0.001 | <0.001 | <0.001 | <0.001 | 0.003 | 0.435 |
| Social | 0.001 | 0.002 | 0.001 | 0.555 | 0.001 | 0.122 |
| Environment | <0.001 | <0.001 | <0.001 | 0.001 | 0.022 | 1.000 |
| QoL | <0.001 | <0.001 | <0.001 | <0.001 | 0.026 | 1.000 |
| Health | <0.001 | <0.001 | <0.001 | <0.001 | 0.004 | 0.630 |

Supplementary Table 6. Mean and 95% confidence intervals of WHOQOL-BREF domain scores stratified by cardiac comorbidities

|  | Heart failure (no) | Heart Failure (yes) | p^1^ | cardiac arrhythmias (no) | cardiac arrhythmias (yes) | p^1^ |
| --- | --- | --- | --- | --- | --- | --- |
| Physical | 77.8 (76.7; 79.0) | 64.3 (58.6; 69.9) | <0.001 | 81.2 (79.9; 82.5) | 70.9 (68.9; 72.8) | <0.001 |
| Psychological | 74.4 (73.4; 75.5) | 68.7 (63.6; 73.8) | 0.038 | 77.2 (76.0; 78.3) | 69.4 (67.7; 71.1) | <0.001 |
| Social | 74.6 (73.3; 75.9) | 72.9 (67.0; 78.7) | 0.571 | 76.5 (74.9; 78.0) | 71.3 (69.2; 73.5) | <0.001 |
| Environment | 77.8 (76.9; 78.8) | 77.9 (73.8; 82.1) | 0.971 | 80.2 (79.2; 81.3) | 74.4 (73.0; 75.9) | <0.001 |
| QoL | 75.5 (74.2; 76.8) | 63.9 (58.2; 69.6) | <0.001 | 77.2 (75.7; 78.8) | 70.5 (68.4; 72.6) | <0.001 |
| Health | 66.6 (65.0; 68.3) | 57.6 (49.4; 65.9) | 0.043 | 71.2 (69.3; 73.1) | 58.7 (56.1; 61.2) | <0.001 |

^1^P-values derived from independent t-tests
